# ASO Author Reflections: Promoting Surgical Science on Social Media

**DOI:** 10.1245/s10434-024-16516-x

**Published:** 2024-11-10

**Authors:** Raja R. Narayan, Syed A. Ahmad, The Annals of Surgical Oncology Social Media Committee

**Affiliations:** 1 Department of Surgery, Loma Linda University, Loma Linda, CA USA; 2 Department of Surgery, University of Cincinnati College of Medicine, Cincinnati, OH USA

## Past

Over two-thirds of physicians have a professional social media presence.^1^ Not only has social media been used to rapidly share journal articles and spur global discussions on emerging topics, recent reports also suggest that increased social media attention can be associated with greater article access and citations.^2^ In response, the *Annals of Surgical Oncology* (ASO) formed a Social Media Committee to leverage X (formerly Twitter) to promote its articles online with the aims of spotlighting publications by the journal and sparking discourse on seminal topics in surgical science. In the 18 months since this committee was formalized, over 50 members have joined across 13 disease topic sections to strategically promote ASO articles through posting video and visual abstracts, brief text and figure summaries, polls on controversial surgical subjects related to a recent publication, and monthly discussions on a featured article called #ASOchat. To evaluate the outcome of these efforts, this study investigated how initiatives by the ASO Social Media Committee impacted the visibility of the journal, publication engagement, and article-level metrics.^3^

## Present

Although there was no difference in the number of manuscripts or the Altmetric scores of articles published in the 18 months before and after formalization of the ASO Social Media Committee, the number of articles downloaded significantly increased in the period after. Additionally, social media posts about articles that received more likes, retweets/reposts, and quote tweets/quotes tended to have increased article downloads. Engagement with social media posts promoting ASO articles increased in the period after the ASO Social Media Committee was formalized with inflections near scheduled #ASOchat events. There were no differences in social media engagement by article type or disease topic section. Overall, formalization of the ASO Social Media Committee likely contributed to increased article engagement on social media and downloads.

## Future

This report highlights the impact formalization of the ASO Social Media Committee had on increasing journal readership as reflected in article downloads and social media engagement. Since more patients and providers are looking online to review clinical recommendations and emerging subjects, respectively, more work is needed to ensure the veracity of these search results. Large internet-based companies, such as Alphabet Inc., have been working with reputable health organizations, such as the National Academy of Medicine, to promote credible resources found on searches of their subsidiary websites, such as Google or YouTube.^4^ In a similar fashion, it will be critical for journals to have an online presence as well to promote their publications broadly and responsibly for an increasingly online audience. Future efforts will center on expanding outreach to multiple social media and networking platforms with a more international and multilingual approach.
